# Personalized Music Recommendation Algorithm Based on Spark Platform

**DOI:** 10.1155/2022/7157075

**Published:** 2022-02-17

**Authors:** Juan Sun

**Affiliations:** Department of Music, Handan University, Handan, Hebei 056005, China

## Abstract

Aiming at the shortcomings of traditional recommendation algorithms in dealing with large-scale music data, such as low accuracy and poor real-time performance, a personalized recommendation algorithm based on the Spark platform is proposed. The algorithm is based on the Spark platform. The K-means clustering model between users and music is constructed using an AFSA (artificial fish swarm algorithm) to optimize the initial centroids of K-means to improve the clustering effect. Based on the scoring relationship between users and users and users and music attributes, the collaborative filtering algorithm is applied to calculate the correlation between users to achieve accurate recommendations. Finally, the performance of the designed recommendation model is validated by deploying the recommendation model on the Spark platform using the Yahoo Music dataset and online music platform dataset. The experimental results show that the use of improved AFSA can complete the optimization of K-means clustering centroids with good clustering results; combined with the distributed fast computing capability of Spark platform with multiple nodes, the recommendation accuracy has better performance than traditional recommendation algorithms; especially when dealing with large-scale music data, the recommendation accuracy and real-time performance are higher, which meet the current demand of personalized music recommendation.

## 1. Introduction

With the continuous development of network and information technology, music works have exploded and gradually formed a vast music work library. Personalized recommendation has become a problem that needs to be solved by powerful music platforms [1, 2]. The recommendation service can filter the music data from the library according to the user's interest and behavior and recommend the corresponding music works intelligently under the premise of meeting the user's personalized needs. A sound recommendation system can save users' time and energy, improve user loyalty, and attract new users and make music platforms more competitive [3–5]. However, because there are many conditions to be met for music recommendation, which leads to the traditional single algorithm recommendation with low accuracy, poor real time, and colossal resource consumption, it cannot meet the current demand for a personalized offer of massive music data.

Based on this, some scholars have proposed improvements and optimization based on the original algorithm models such as collaborative filtering, content, and model and have achieved certain achievements. Lampropoulou et al. [6] proposed a music recommendation system based on SVM to accomplish the query of the same type of music resources through music content retrieval and collaborative filtering algorithm to achieve personalized music recommendation. However, the effect of recommendation capability is not satisfactory when dealing with large-scale dimensional data. Tian et al. [7] proposed a hybrid LX recommendation algorithm by integrating the logistic regression method and XG-Boost (eXtreme Gradient Boosting). It verified the effectiveness of the LX model by real music dataset, and experiments showed that the method has high accuracy. The experiments show that the method has high recommendation accuracy. However, due to the high spatial complexity of the XG-Boost preordering process, the implementation process is more complicated, and the computational resources are more consumed. The real-time recommendation capability is not high. Using k-means clustering to find out users with similar attributes and pLSA (probabilistic Latent Semantic Analysis) technology to obtain the type of music users often listen to, the algorithm combines to achieve accurate recommendations with high efficiency [8] The accuracy and efficiency are high.

Although the above recommendation algorithm can accomplish music recommendations well, there is still a large room for improvement. Therefore, this paper proposes a K-means clustering music recommendation method based on AFSA optimization based on the Spark platform. The improved AFSA is used to solve the global optimization-seeking and local extremum problems of the K-means algorithm to improve the accuracy of multicategory clustering. The collaborative filtering algorithm is also used to deal with the similarity between users and improve the clustering effect and recommendation efficiency by the parallel computing capability of multiple nodes of the Spark platform.

## 2. Spark Platform Introduction

**Figure 1:**
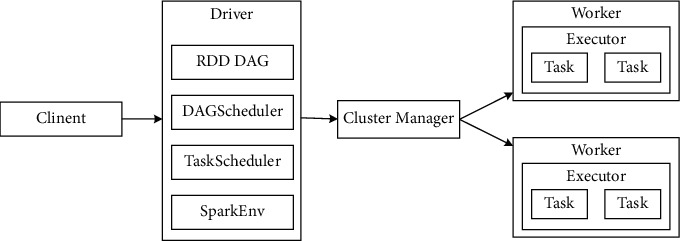
Traditional character recognition process.

Apache Spark is a distributed computing engine specialized in processing large-scale data, with the advantages of fast data processing, strong stability, and scalability and support for multinode collaboration, which can complete data stream processing, interactive query, and batch processing in real time [9, 10]. As shown in Figure 1, Spark adopts the management model of master-slave nodes, and the nodes have the same computing capability in terms of functional structure. It is responsible for controlling the executor or driver process to complete resource management and task scheduling. The executor refers to the process of an application running on the work node.

Spark platform uses DAG (directed acyclic graph) job scheduling scheme. The dependencies between different data are specified by building a model of data relationships in RDDs (resilient distributed datasets). To facilitate the iterative computation of data, the results of the distributed analysis are usually stored in memory. The RDD is a read-only, partitioned, and immutable dataset. Since Spark uses the RDD computation model to do most of the data computation, the model calls more methods and does not need to focus on the underlying scheduling details [11]. This fundamentally avoids the problem of low computing efficiency caused by multiple iterations in the clustering process, dramatically improves the effect of data clustering and recommendation, and meets the demand for real-time and intelligent music recommendations.

## 3. K-Means Clustering

**Figure 2:**
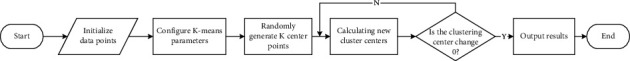
Flow of K-means algorithm.

K-means clustering is one of the commonly used algorithms in data mining. The algorithm uses the distance between data sample points to measure similarity. The closer the distance, the higher the similarity of the data. Set up a dataset *C* = {*c*_*i*_, *i* = 1, 2,…, *m*} with *m* sample points; each sample has an *N* data dimension feature description [12]. The K-means algorithm aims to allocate all the data samples in the *n* dataset and divide the pieces into corresponding *K* clusters according to the principle of similarity, cluster *D* = {*d*_*k*_, *k* = 1, 2,…, *K*}. The error of clustering is evaluated using the sum of squared errors, which is computed repeatedly until the sum of squared errors of clustering is the smallest [13]. The algorithm flow is shown in Figure 2.

There are more methods to calculate the distance between data sample points, and the K-means algorithm mainly uses the Euclidean distance to measure the similarity between data samples. The Euclidean distance between two data samples in dataset *C* can be expressed as [14]

(1) $$\begin{array}{c} \text{dist}\left(x,y\right)=\sqrt{\overset{n}{\underset{i=1}{\sum }}{\left(x_{i}-y_{i}\right)}^{2}}. \end{array}$$

The mean of the *N* data samples within cluster *d*_*k*_ is calculated and considered as the center of mass *h*_*k*_ of the cluster, and the expression is

(2) $$\begin{array}{c} h_{k}=\frac{\sum_{c_{i}\in d_{k}}c_{i}}{N}. \end{array}$$

The criterion for clustering sum of the squared errors G as data samples:

(3) $$\begin{array}{c} G=\overset{K}{\underset{k=1}{\sum }}\underset{c_{i}\in d_{k}}{\sum }\left|c_{i}-h_{k}\right|. \end{array}$$

The implementation of the K-means algorithm is relatively simple. Still, the number of clusters depends on manual experience setting, which takes a long time when dealing with large-scale data such as music and video, and the accuracy of clustering is not ideal. Moreover, the initial clustering centroid of the algorithm is selected randomly, which is prone to the situation that the clustering results vary greatly. If a relatively isolated point is selected as the initial clustering centroid, it will directly affect the accuracy and efficiency of clustering [15, 16]. Considering the actual application requirements, the K-means algorithm needs to be improved to enhance the clustering accuracy and efficiency.

## 4. Improved Artificial Fish Swarm Algorithm

### 4.1. Artificial Fish Swarm Algorithm

**Figure 3:**
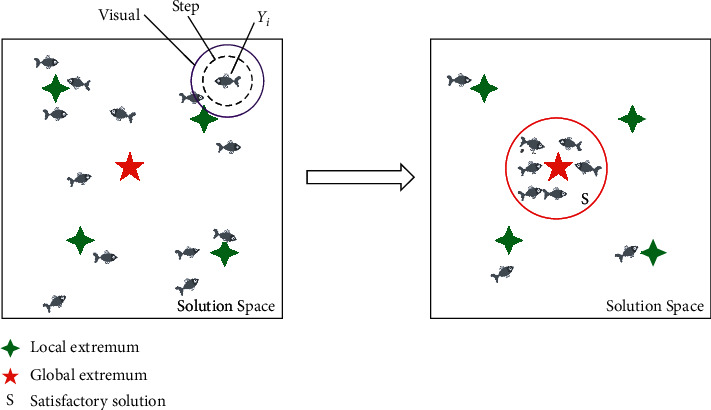
Principle diagram of artificial fish seeking advantage.

AFSA is a bionic optimization algorithm proposed by studying the intelligent behavior of fish. Based on the environmental stimulus information received by the fish, an artificial fish is constructed, and its feeding, gathering, and tailing behaviors are simulated to accomplish automatic optimization of the solution space [17, 18]. In this paper, AFSA is used to select the initial cluster center of mass for K-means clustering. The data sample of the desired cluster is used as the artificial fish. When the fish in the group find food, the other individuals in the collection adjust their positions according to their state and food position and finally obtain the optimal global value, which is the initial center point of the cluster. The principle of artificial fish seeking is shown in Figure 3.

Let *Y*_*i*_(*t*) be the spatial position of the artificial fish *i* at the *t* moment; Δ*Y*_*i*_(*t* + 1) denotes the change in spatial part of the fish *i* at the *t* + 1 moment compared to the *t* moment; then, the random movement behavior of the artificial fish *Y*_*i*_ can be expressed as

(4) $$\begin{array}{c} \Delta Y_{i}\left(t+1\right)=\text{Rand}*\text{Step}*\left[Y_{i}\left(t+1\right)-Y_{i}\left(t\right)\right], \end{array}$$

where Rand() is a random function and *Step* denotes the update of the artificial fish stocks. Then, a specific state *Y*_*j*_ within the visual of the artificial fish *Y*_*i*_ is randomly selected as

(5) $$\begin{array}{c} Y_{j}=Y_{i}+\text{Visuanl}*\text{Rand}. \end{array}$$

Calculate the objective function values *Z*_*i*_ and *Z*_*j*_ for *Y*_*i*_ and *Y*_*j*_. If *Z*_*j*_ is better than *Z*_*i*_, then *Y*_*i*_ is one step ahead in the direction of *Y*_*j*_. There are many kinds of filtering algorithms:

(6) $$\begin{array}{c} Y_{i}^{t+1}=Y_{i}^{t}+\frac{Y_{j}-Y_{i}^{t}}{\left\|Y_{j}-Y_{i}^{t}\right\|}*\text{Step}*\text{Rand}. \end{array}$$

If *Z*_*j*_ is worse than *Z*_*i*_, fish *Y*_*i*_ reselects another state *Y*_*j*_ in its field of view and determines whether it can advance. After repeated attempts of Tray-number times, if it still cannot get the condition to satisfy the advancement, the random behavior is executed.

Let the food concentration function in spatial coordinates be *f*(*y*), the coordinate food concentration be *f*_max_, and the food concentration at the *Y*_*i*_ coordinate of the artificial fish be *f*(*Y*_*i*_). Based on the maximum value of the food concentration *f*(*y*) within the field of view of the fish, determine whether the food location *Y*_*o*_ is in the visible range of the fish, and if it is visible, perform the trailing behavior:

(7) $$\begin{array}{c} Y_{i}^{t+1}=Y_{i}^{t}+\frac{Y_{O}-Y_{i}^{t}}{\left\|Y_{O}-Y_{i}^{t}\right\|}*\text{Step}*\text{Rand}. \end{array}$$

### 4.2. Optimization of Artificial Fish Swarm Algorithm

#### 4.2.1. Optimization of Fish School Behavior

In the foraging behavior of AFSA, *Y*_*j*_ is any state within the current field of view of the artificial fish. The artificial fish performs random behavior when the selected form cannot satisfy the forward condition. The existence of unexpected behaviors in the algorithm will result in lower accuracy of the foraging results; the convergence process of the algorithm in the later stage is prone to the phenomenon of artificial fish searching in a roundabout way at the global extremum, which leads to invalid calculation. At the same time, when the artificial fish fails in clustering and tail-chasing behavior, it performs foraging behavior, and the artificial fish has more invalid forward behavior, which makes the convergence time of the algorithm longer and the iterative computation more significant. Therefore, this paper optimizes the fish swarming behavior in the original algorithm in the following two aspects to further improve the algorithm's performance.

(1) When the foraging behavior fails, the artificial fish *Y*_*i*_ moves one step forward in the direction of the relatively better state in the bulletin board record. The optimized expression for the foraging behavior is
  (8) $$\begin{array}{c} Y_{i}^{t+1}=Y_{i}^{t}+\left[Y_{\text{better}}^{t+1}-Y_{i}^{t}\right]*\text{Step}*\text{Rand}, \end{array}$$
where *Y*_better_ is the relatively better state in the bulletin board record and [*Y*_better_^*t*+1^ − *Y*_*i*_^*t*^] is the increment of that state.
The optimized foraging behavior discards the phenomenon of artificial fish randomly advancing and provides a better possibility of running for fish movement, which can effectively avoid artificial fish falling into local optimum and can further improve the accuracy of the search results.
(2) When the clustering behavior or tail-chasing behavior fails, the artificial fish no longer performs the foraging operation but executes the random swimming behavior, and the optimized unexpected moving behavior is defined and expressed as
  (9) $$\begin{array}{c} Y_{i}^{t+1}=Y_{i}^{t}+\text{Step}\left(t+1\right). \end{array}$$

By optimizing the forward rule after the failure of clustering and tailing behavior, we can reduce the invalid brash behavior of artificial fish, thus effectively reducing the algorithm running time and fundamentally reducing the computational complexity.

#### 4.2.2. Adaptive Field of View and Step Size

The two parameters of the artificial fish's field of view and step length determine the current search range and the subsequent moving speed. When artificial fish are searching far away from the extreme point, they should have a larger field of view and step to improve search speed. Also, when the fish in the area nearer to the extreme value point are searched, the values of the field of view and step length should be small to ensure the accuracy of the search. Considering the global fixed values of the field of view and step length in the original algorithm, it is easy to fall into the dire situation of blind search [19, 20]. In this paper, the adaptive value of both is accomplished by introducing the decay factor.

(10) $$\begin{array}{c} \sigma =\frac{Y_{j}-Y_{i}}{\left\|Y_{j}-Y_{i}\right\|}*\text{Rand}, \end{array}$$

where *σ* is the attenuation factor with forwarding direction to constrain the step length, the field of view, and forward movement of the artificial fish, *σ* ∈ [0,1]. Then, the equations of adaptive step length and adaptive decay field of view can be expressed as

(11) $$\begin{array}{c} \text{Step}\left(t+1\right)=\text{Step}\left(t\right)*\sigma ,\\ \text{Visual}\left(t+1\right)=\text{Visual}\left(t\right)*\sigma . \end{array}$$

The iterative process of AFSA is a continuous process of artificial fish aggregation to the global extremum. By introducing a decay factor, the fish can dynamically adjust the field of view and step size according to the external environmental information during the search process. In the early stage of the algorithm's operation, the artificial fish use a larger field of view and step size to conduct a coarse search in a wide range near the optimal value, thus speeding up the individual search speed and making the algorithm converge quickly. After several iterations, the artificial fish gradually approaches the extreme value. At the end of the iteration, the field of view and step size is adjusted adaptively by the attenuation factor to enhance the local search ability and reduce the oscillation range. The artificial fish can quickly find extreme global points with high accuracy. Especially in the case of multidimensional data, the algorithm of finding the optimal effect with the introduction of the decay factor is more prominent.

### 4.3. User-Based Collaborative Filtering

After clustering by the AFSA-optimized K-means algorithm, the user's rating matrix of all resources and services to be recommended can be obtained. The collaborative filtering algorithm can find users with the same rating items in this rating matrix and calculate the similarity to complete practical personalized music recommendations. There are many kinds of filtering algorithms, but PPMCC (Pearson product-moment correlation coefficient) solves the difference of each dimension, and the similarity calculation process focuses on the whole so that it can complete the multidimensional examination of the same user [21]. So, this paper uses PPMCC to calculate the similarity between users. The expression is

(12) $$\begin{array}{c} \text{sim}\left(u_{u},u_{v}\right)=\frac{\sum_{i_{c}\in I\left(u,v\right)}\left(r_{uc}-{\bar{r}}_{u}\right)\left(r_{vc}-{\bar{r}}_{v}\right)}{\sqrt{\sum_{i_{c}\in I\left(u,v\right)}{\left(r_{uc}-{\bar{r}}_{u}\right)}^{2}}\times \sqrt{\sum_{i_{c}\in I\left(u,v\right)}{\left(r_{vc}-{\bar{r}}_{v}\right)}^{2}}}, \end{array}$$

where *I(u*, *v)* represents the set of shared items jointly rated by users *u*_*u*_ and *u*_*v*_, *r*_*uc*_ and *r*_*vc*_ represent the ratings of items *i*_*c*_ by users *u*_*u*_ and *u*v, respectively, and ${\bar{r}}_{u}$ and ${\bar{r}}_{v}$ represent the average ratings of all calculated items by users *u*_*u*_ and *u*v, respectively.

Table 1 shows the user-user similarity matrix W calculated by the PPMCC.

### 4.4. Music Recommendation Process Based on Spark

**Figure 4:**
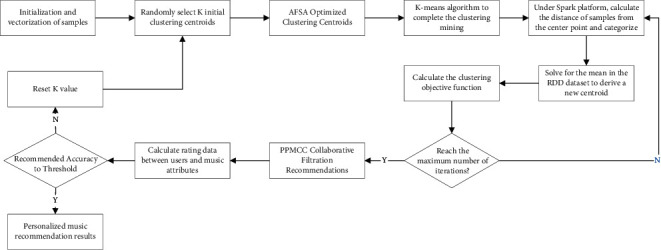
Personalized music recommendation algorithm flow based on Spark platform.

The recommendation process of K-means clustering mining based on AFSA optimization is shown in Figure 4. First, the requirement analysis is performed for the music personalization recommendation task. The Spark platform deploys distributed nodes of a suitable size to build the K-means clustering operation model. The AFSA algorithm is applied to optimize the centroids of the initial K-means clustering and obtain the clustering results. According to the scoring model between users and users and users and music attributes, the collaborative filtering algorithm calculates the similarity between users. In turn, the personalized recommendation of music resources is completed.

## 5. Experiment

### 5.1. Experimental Dataset and Clustering Center K Values

To verify the performance of the AFSA-optimized K-means clustering personalized music recommendation algorithm on the Spark platform, public and proprietary datasets are tested separately. The public dataset is Yahoo Music, a classical dataset for testing recommendation systems which can well validate the recommendation performance of clustering mining. The dataset contains large-scale user and music resource data to validate the recommendation system's performance adequately. The experimental dataset is shown in Table 2. The Spark platform contains one master node and nine work nodes with the same hardware configuration, CPU: I5-11400: memory: Kingston 3600 8gb ∗ 2; and system: IOP 4.2 Version, including Spark Version 1.61.

The clustering center K value is more sensitive to the music scoring matrix and has a more significant impact on the stability of collaborative filtering recommendations. Therefore, this paper differentiates the K values and uses RMSE (root mean squared error) as an evaluation index to verify the recommendation accuracy under different K values [22]. The expression of RMSE is

(13) $$\begin{array}{c} \text{RMSE}=\sqrt{\frac{\sum_{b=1}^{N}{\left(p_{b}-q_{b}\right)}^{2}}{N}}, \end{array}$$

where *p*_*b*_ denotes the predicted score, *q*_*b*_ denotes the actual score, and *N* represents the number of items. Smaller RMSE values indicate better algorithm performance.

**Figure 5:**
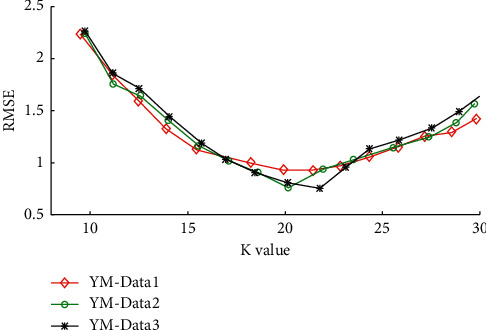
Recommended accuracy RMSE values for different *K* values.

The data from the Yahoo Music dataset were randomly taken and formed into three sample sets of different sizes YM-Data1 (13.82 MB), YM-Data2 (43.39 MB), and YM-Data3 (140.24 MB), and then experiments were conducted with the three sample data, respectively, to derive different K-value recommendation accuracy RMSE values, as shown in Figure 5.

As shown in Figure 5, the RMSE value decreases and increases as the K value increases, and the RMSE value is optimal for the three sample sets of YM-Data1, YM-Data2, and YM-Data3 when the *K* value is 20, 20, and 22. When increasing the K values, the RMSE values gradually become more extensive, and the recommendation stability worsens. Therefore, in the subsequent training process, the range of *K* values is set to [20, 22].

### 5.2. Performance of the Recommendation Algorithm

#### 5.2.1. Public Datasets

The K value is set to 20, and the designed A-optimized K-means algorithm is used for clustering mining. The recommendation algorithm model is used for training the Yahoo Music sample set. All master nodes and work nodes are involved in the computation during training. The recommended performance of the three sample sets is shown in Table 3.

As shown in Table 3, the difference in sample set size has little influence on the recommended usage time and accuracy, and it has good real-time recommendation ability because the data are loaded into the distributed memory of the Spark cluster host and the conversion iteration completes faster, especially when the sample data size is small.

#### 5.2.2. Online Music Dataset

**Figure 6:**
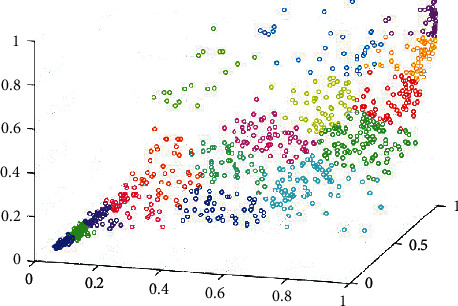
Visualization of clustering results of KW-Data3.

To further validate the recommended performance of this method, a dataset from an online music platform is used for testing. First, four sets of sample sets with different capacities based on music platform are built: KW-Data1 (10.75 MB), KW-Data2 (474.84 MB), KW-Data3 (1.62 GB), and KW-Data4 (6.65 GB). Then, cluster mining is applied to each data sample to get the score between all users and music resource attributes. Finally, the recommended results are obtained by using the collaborative filtering algorithm. KW-Data2 is used as an example to visualize its clustering results to facilitate the observation of the clustering effect, and the result is shown in Figure 6.

To make the results comparable, the algorithm performance was experimented with differentially setting the number of clustering centers for K taking values of 18, 20, and 22. The obtained results are shown in Table 4.

As can be seen from Table 3, the accuracy rate stays above 90% when K values are selected from the optimization algorithm, and the recommendation accuracy rate improves by 20.3%–21.7% compared with the randomly selected K values. Since the Spark platform uses multinode computing, the recommendation time gradually increases with the increase of sample capacity under different K values. Still, there is no rapid growth phenomenon, in line with the actual rule.

### 5.3. Spark Platform Acceleration Performance Testing

The higher the speedup ratio is, the more parallelized the platform is. The acceleration ratio expression is

(14) $$\begin{array}{c} \text{Speed up}\left(\rho \right)=\frac{T_{1}}{T_{\rho }}, \end{array}$$

where *T*_*1*_ denotes the recommended time for a single node and *T*_*ρ*_ denotes the time used for multinode parallel computation under the Spark platform. The speedup performance at the different number of nodes is shown in Table 5.

From Table 5, we can see that the more the number of worker nodes is, the more significant the speedup effect is due to the excellent parallelization capability of Spark platform, and the larger the volume of the sample set, the more influential the impact of the number of nodes on the speedup ratio.

### 5.4. Performance Comparison of Different Recommendation Algorithms

**Figure 7:**
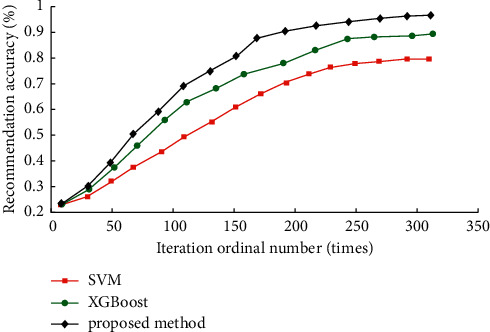
Recommendation performance of different algorithms.

Five hundred samples were randomly selected from each online music platform sample set. A new selection set KW-Data5 was constructed to test the SVM classification algorithm proposed in [6], the XG-Boost algorithm presented in [7], and the algorithm in this paper to ensure the comparability of the algorithms and further verify the performance of the optimized K-means clustering based on the improved artificial fish swarm algorithm. The recommendation performance of the three algorithms is shown in Figure 7.

As can be seen from Figure 7, the recommendation accuracy of each algorithm increases with the number of iterations and then gradually stabilizes. However, the recommendation accuracy of this method reaches 96.23%, which is 6.77% and 15.32% higher than that of XG-Boost and SVM, respectively, and the recommendation performance is more excellent. The convergence speed of different algorithms: the method in this paper tends to be stable after 245 iterations; the SVM tends to be stable after 230 iterations; XG-Boost algorithm completes convergence after 295 iterations, which is slower. Therefore, based on the comprehensive recommendation performance comparison results, it can be concluded that the improved AFSA-based optimized K-means clustering method has good recommendation ability, can better balance the relationship between accuracy and convergence speed, and is suitable for handling large-scale personalized music recommendation work.

## 6. Conclusion

To address some shortcomings of the traditional music recommendation algorithm, the improved AFSA is applied to solve the global optimization and local extremum problems of the K-means algorithm, thus improving the effect of multicategory clustering, completing the personalized recommendation of music by collaborative filtering algorithm, and improving the speed of large-scale data processing by using the parallelized computing feature of Spark distributed platform. The experimental results show that the AFSA-optimized K-Means clustering mining algorithm has good recommendation capability, and the recommendation accuracy is improved by 6.77% and 15.32% compared with the commonly used SVM and XG-Boost methods; the algorithm iterates faster to meet the requirements of real-time recommendation, which is enough to improve the recommendation efficiency of personalized music effectively and can be used as a reference for music, video, and similar fields, with good guiding significance.

## Figures and Tables

**Table 1:** User-user similarity matrix W.

*U*	*U*			
	*u*_*1*_	*u*_*2*_	…	*u*_*m*_
*u*_*1*_	sim (*u*_*1*_, *u*_*1*_)	sim (*u*_*1*_, *u*_*2*_)	…	sim (*u*_*1*_, *u*_*m*_)
*u*_*2*_	sim (*u*_*2*_, *u*_*1*_)	sim (*u*_*2*_,*u*_*2*_)	…	sim (*u*_*2*_, *u*_*m*_)
…	…	…	…	…
*u*_*m*_	sim (*u*_*m*_, *u*_*1*_)	sim (*u*_*m*_, *u*_*2*_)	…	sim (*u*_*m*_, *u*_*m*_)

**Table 2:** Information on the datasets used for the experiments.

Dataset	Number of users	Number of works	Number of ratings	Rating density	Rating level
Yahoo Music	7400	3000	15335	0.0006	1, 2,…, 100
Online music platform	11406	34668	170321	0.0152	1, 2,…, 10

**Table 3:** Recommendation performance based on Yahoo Music dataset.

Sample set	Accuracy (%)			Recommended time (s)
	Minimum value	Average value	Maximum value	
YM-Data1	82.872	85.097	87.233	0.973
YM-Data2	82.165	85.714	87.792	0.944
YM-Data3	83.281	85.639	87.935	0.984

**Table 4:** Recommended performance for different *K* values.

Sample set	Capacity	*K* = 18		*K* = 20		*K* = 22	
		Accuracy (%)	Recommended time (s)	Accuracy (%)	Recommended time (s)	Accuracy (%)	Recommended time (s)
KW-Data1	10.75 MB	84.407	3.375	93.524	1.273	93.177	1.451
KW-Data2	474.84 MB	81.762	7.243	93.007	3.104	93.090	3.329
KW-Data3	1.62 GB	80.155	9.716	91.808	4.774	91.832	4.808
KW-Data4	6.65 GB	70.094	19.458	90.450	8.635	90.892	8.924

**Table 5:** Comparison of acceleration performance with different number of nodes.

Sample set	Capacity	Number of work nodes (pcs)	Speedup	Sample set	Capacity	Number of work nodes (pcs)	Speedup
KW-Data1	10.75 MB	1	1.000	KW-Data2	474.84 MB	1	1.000
		5	1.002			5	1.007
		10	1.003			10	1.015
KW-Data3	1.62 GB	1	1.000	KW-Data4	6.65 GB	1	1.000
		5	5.486			5	19.775
		10	12.837			10	48.093

## Data Availability

The data used to support the findings of this study are available from the corresponding author upon request.

## References

[1] Hung-Yi Lo H. Y., Ju-Chiang Wang J. C., Hsin-Min Wang H. M., Shou-De Lin S. D. (2011). Cost-sensitive multi-label learning for audio tag annotation and retrieval. *IEEE Transactions on Multimedia*.

[2] Bae J., Kim J. (2019). Deep learning music genre automatic classification voting system using softmax. *Journal of the Korea Institute of Information and Communication Engineering*.

[3] Yudiana W. A., Ariyanti M., Alamsyah A. Wisdom of the crowd” as personalized music recommendation model for langit musik service.

[4] Xu D., Dai Y., Dai W. Smart recommendation of personalized cloud music service based on mental model.

[5] Geng J. (2021). Personalized analysis and recommendation of aesthetic evaluation index of dance music based on intelligent algorithm. *Complexity*.

[6] Lampropoulou P. S., Lampropoulos A. S., Tsihrintzis G. A. (2009). A mobile music recommender system based on a two-level genre-rating SVM classifier enhanced by collaborative filtering. *New Directions in Intelligent Interactive Multimedia Systems and Services - 2*.

[7] Tian H., Cai H., Wen J., Li S., Li Y. A music recommendation system based on logistic regression and eXtreme gradient boosting.

[8] He L., He K. (2022). Efficient memory-bounded optimal detection for GSM-MIMO Systems,’’ to appear. *IEEE Transaction Communication*.

[9] Yang F., Wang H., Fu J. (2021). Improvement of recommendation algorithm based on collaborative deep learning and its parallelization on Spark. *Journal of Parallel and Distributed Computing*.

[10] Singh P., Dutta K., Kaye R., Garg S. Music listening history dataset curation and distributed music recommendation engines using collaborative filtering.

[11] Lai X., Fan L. (2022). Outdated access point selection for mobile edge computing with cochannel interference,” to appear. *IEEE Transaction Vehicular Technology*.

[12] Cai Z., Fu L., Li W. (2021). Research and analysis of music development based on k-means and PCA algorithm. *Journal of Physics: Conference Series*.

[13] Jung Y. M., Whang J. J., Yun S. (2020). Sparse probabilistic K-means. *Applied Mathematics and Computation*.

[14] Azcarraga A., Flores F. K. A study on self-organizing maps and K-means clustering on a music genre dataset.

[15] Foleis J. H., Tavares T. F. (2020). Texture selection for automatic music genre classification. *Applied Soft Computing*.

[16] Sitompul B. J. D., Sitompul O. S., Sihombing P. (2019). June). Enhancement clustering evaluation result of davies-bouldin index with determining initial centroid of k-means algorithm. *Journal of Physics: conference Series*.

[17] Lu J., Tang M. (2022). Analytical offloading design for mobile edge computing based smart internet of vehicle. *To Appear in EURASIP Journal on Advances in Signal Processing*.

[18] Zhao J., Fang Y., Hong Q., Pan Z., Huang L., Zhang D. UAV-based identification of achnatherum splendens community combining K-means and artificial fish swarm algorithm.

[19] Zhang L., Zhou X., Fei T. (2019). Research of improved artificial fish swarm portfolio optimization algorithm based on adaptive levy mutation. *Journal of Internet Technology*.

[20] Peng J., Wan Z., Wei Q., Jiang H., Wei S. (2018). An improved artificial fish swarm algorithm. *International Journal for Engineering Modelling*.

[21] Patil A. E., Patil S., Singh K., Saraiya P., Sheregar A. (2019). Online book recommendation system using association rule mining and collaborative filtering. *International Journal of Computer Science and Mobile Computing*.

[22] Zhang L., Zhu F. (2022). DQN based mobile edge computing for smart internet of vehicle. *To Appear in EURASIP Journal on Advances in Signal Processing*.

